# Effects of different types of Advance Care Planning workshops: A scoping review protocol

**DOI:** 10.1371/journal.pone.0322661

**Published:** 2025-05-20

**Authors:** Liu Yang, Cui Su, Xianlin Wang, Mei Chen, Renli Deng

**Affiliations:** 1 Department of Emergency, Affiliated Hospital of Zunyi Medical University, Zunyi, Guizhou Province, China; 2 School of Nursing, Zunyi Medical University, Zunyi, Guizhou Province, China; Polytechnique Montreal, CANADA

## Abstract

**Introduction:**

Advance Care Planning(ACP) plays a crucial role in ensuring that patients’ wishes are respected and fulfilled, helping to improve the quality of care and the efficient use of resources. Achieving effective ACP promotion depends on appropriate education and training. Evidence exists that ACP workshops are feasible and effective in enhancing the understanding, competence, confidence, and engagement in ACP among trainees. Systematic summary and analysis of existing ACP workshop-related research will help identify knowledge gaps and research trends in the current field and provide a standardized framework and empirical support for future training design.

**Objectives:**

The overall aim of this scoping review was to systematically map existing literature about ACP workshops, provide an overview of the contents and effects of various ACP workshops, and identify limitations and gaps in knowledge.

**Methods:**

This scoping review will adhere to the JBI methodology and the results will reported according to the PRISMA-ScR guidelines. Relevant articles will be systematically searched in six electronic databases (Pubmed, PsycINFO, CINAHL, Scopus, Web of Science, and Google Scholar). The research on ACP workshops conducted in any population will be included. Two reviewers individually selected the studies: first by screening titles and abstracts and second by screening full-text articles. A third reviewer will arbitrate discrepancies during the screening process. Extract data in a standardized manner using forms that have undergone team review, and data will then be synthesized and interpreted.

**Discussion:**

This scoping review will identify effective training methods, topics, and research gaps by comparing the content and effectiveness of different workshops, which will help to form best practices and enhance the effectiveness of ACP training.

REGISTRATION DETAILS: Open Science Framework under https://doi.org/10.17605/OSF.IO/2ZUP6.

## Introduction

Advance Care Planning(ACP) represents a patient-centered, voluntary, and continuous process, enabling individuals to articulate their values, goals, and preferences for future medical treatments with family, healthcare providers, and relevant stakeholders, particularly when they are of sound mind and capable of making informed decisions [1–3]. This process underscores the importance of dialogue among patients, their family, and healthcare professionals to reach consensus on healthcare choices, including clarifying values, appointing decision-makers, and finalizing pertinent documents [3]. ACP aims to honor patient autonomy, enhance care quality, strengthen relationships, prepare for end-of-life scenarios, and prevent overtreatment [4]. Research has consistently shown that ACP leads to positive outcomes for patients, their families [5], healthcare professionals [6], and the overall healthcare system [7]. For instance, Goswami et al. [8] demonstrated that ACP plays a crucial role in helping cancer patients comprehend their condition and prognosis, aiding them in making end-of-life care decisions. Liu et al. [5] also showed that ACP also enhances the alignment of end-of-life care between patients and caregivers, boosts caregivers confidence in decision-making, and improves communication quality during this critical period. In addition, it supports older adults in achieving goal-aligned care [9], resulting in reduced hospitalizations, less intensive end of life interventions, decreased medical expenses, and optimized resource utilization [7].

In current clinical practice, the significance of ACP as a pivotal strategy to enhance the quality of care for patients at the end of life has been widely acknowledged. In the process of implementing Advance Care Planning (ACP), we face numerous challenges that not only involve the perceptions and attitudes of patients, families, and healthcare providers, but also encompass institutional, cultural, and legal barriers [10–14]. The apparent deficiencies in understanding and effective implementation of ACP among patients, families, and healthcare providers can be largely attributed to the lack or inadequacy of ACP training [11–12]. As the healthcare environment continues to evolve and patient needs become increasingly complex, addressing these challenges requires comprehensive training programs for healthcare providers and educational programs for patients and families to ensure that ACP becomes an integral part of high-quality healthcare services.

Workshops have emerged as a widely adopted method to raise awareness about the importance of ACP and to promote the development of relevant skills [15–16]. These interactive learning and training sessions, typically conducted in small groups, are designed to foster skill development and learning through hands-on activities and participant engagement [15–16]. By engaging participants in practical exercises and discussions, workshops effectively enhance understanding of ACP and equip individuals with the necessary skills to implement it effectively. Evidence suggests that ACP workshops are effective in enhancing participants’ understanding, skills, confidence, and engagement with ACP, promoting broader implementation [17–20]. Such workshops have successfully increased multidisciplinary staff’s knowledge, self-efficacy, attitude, and comfort related to ACP discussions, while also addressing cultural and spiritual concerns [17–18]. Community-led, peer-facilitated ACP workshops have been found to improve public knowledge, motivation, and confidence in participating in ACP, ultimately enhancing their involvement [19]. Role-playing ACP workshops, specifically, assist students in recognizing the impact of high-quality cross-professional dialogues on the care patients receive [20].

Despite numerous studies showing the effectiveness of ACP workshops, there is still a knowledge gap in comprehensively evaluating and comparing different workshop formats and contents. Research on the most effective formats and contents for improving clinical practice remains inadequate. Therefore, a systematic comparison and comprehensive evaluation of ACP workshops is necessary. This necessity arises from the diversification of workshops, which cater to various target groups and focus on different skill enhancements, making it hard to identify the best approaches [20–22]. Consequently, decision-makers, researchers, or funders face difficulties in ascertaining the most effective design, thereby impeding informed decision-making or resource allocation. Additionally, variability in assessment standards across studies hinders result comparison [23], which may reduce the reusability of training resources. Finally, cultural and regional differences can influence workshop effectiveness and acceptance [24], which hinders the accumulation and dissemination of knowledge.

In summary, this study aims to fill this gap by systematically mapping ACP workshop literature, identifying employed methods, and assessing their effectiveness through a scoping review. This will provide insights into the most effective workshop formats for different target groups and skills, guiding future workshop design and implementation.

## Objectives

This scoping review aims to systematically map existing literature about ACP workshops, provide an overview of the contents and effects of various ACP workshops, and identify limitations and gaps in knowledge to provide direction for improving and optimizing ACP training programs. To best address this, we will answer four sub-questions: (1) How many types of ACP workshops (differentiated by research populations, training content and location, etc.)?, (2) What are the characteristics of various ACP workshops (including but not limited to their purpose, target population, venue, training content, and learning resources)?, (3) What are the main outcomes of various ACP workshops? (4) What are the limitations and gaps in current literature?

## Methods

### Protocol design

A scoping review is a knowledge synthesis method aimed at determining the range of available evidence on a specific topic and visually presenting this evidence through mapping [25]. This scoping review will guided by the Joanna Briggs Institute(JBI) published methodological guidance [26–28], leveraging the population, concept, and context (PCC) framework [27] to construct the research question and determine the inclusion and exclusion criteria. To ensure the rigor and transparency of our scoping review, we will adhere strictly to the guidelines and checklist provided by PRISMA-ScR (Preferred Reporting Items for Systematic Reviews and Meta-Analyses Extension for Scoping Reviews) [28–29]. Furthermore, we will also utilize the PRISMA-P checklist [30] to guide the preparation and reporting of our protocol. The review protocol is registered at Open Science Framework under https://doi.org/10.17605/OSF.IO/2ZUP6.

### Step 1: Research question

Various scholars have developed multiple types of ACP workshops to facilitate ACP communication. However, there needs to be a more systematic comparison and comprehensive analysis of the contents and effects of these diverse workshops. The scoping review aims to systematically map existing literature on ACP workshops to answer the following research questions (RQs):

RQ1: How many types of ACP workshops (differentiated by research populations, training content and location, etc.)?
RQ2: What are the characteristics of various ACP workshops (including but not limited to their purpose, target population, venue, training content, and learning resources)?
RQ3: What are the main outcomes of various ACP workshops?
RQ4: What are the limitations and gaps in current literature?

### Step 2: Identifying relevant literature

The literature search strategy will be conducted in three phases [27]:

Phase 1: A preliminary search of the PubMed database will be performed in August 2024 to gather relevant literature. Co-authors will then analyze the titles, abstracts, and index terms extracted from this initial search to identify potential search terms including “Advance Care Planning”, “Communication”, “Workshop”, “Role-Play”, “Primary Clinicians”, “Students”, “Adults”.

Phase 2: Following the initial search, the research team will engage in detailed discussions with information specialists. After collaborative deliberation, we have devised a comprehensive search strategy to retrieve literature closely related to the topic of ACP workshops. The strategy employs core search terms such as ‘advance care planning’ and ‘workshop’ along with their synonyms and related phrases, and combines MeSH terms with free-text keywords to ensure the comprehensiveness and depth of the search.

Phase 3: Based on the search findings in Phase 2, the author will conduct the main search and subsequently be peer-reviewed by another co-author according to the Peer Review of Electronic Search Strategies checklist [31]. The research team, who have undergone systematic training in evidence-based nursing, will target six electronic databases: Pubmed, PsycINFO, CINAHL, Scopus, Web of Science, and Google Scholar. For PubMed, a detailed search strategy is provided as an example in S3 Table. The search strategies for the other five databases will be adjusted according to their unique characteristics and functionalities to ensure optimal retrieval of relevant literature. The search scope is set from the establishment of the databases to November 2024 for all databases. No language or other restrictions will be applied in the literature search. However, to ensure the quality and reliability of the retrieved literature, gray literature such as unpublished reports, conference proceedings, and dissertations will not be included in this search strategy. We expect to complete the search processes in December 2024.

To ensure comprehensiveness, the reference lists of the included articles will be examined to identify any potentially overlooked studies. Additionally, the reference lists of pertinent reviews will be reviewed, and any relevant primary studies discovered will be manually incorporated.

### Step 3: Study selection

We will include all peer-reviewed sources of quantitative, qualitative, or mixed-method research but not include systematic reviews, narrative reviews, meta-analyses, bibliometric analyses, letters, case studies, books or book chapters, comments, editorials, congress abstracts or symposia proceedings, poster presentations, and dissertations. Eligibility criteria will defined according to the population, concept, and context framework [27]. Studies will be considered eligible according to the criteria described below:

#### Population.

All populations, including but not limited to (1) Healthcare professionals (doctors, nurses, social workers, etc.), (2) Medical students and interns, (3) General adult public, (4) Community residents.

#### Concept.

(1) ACP workshop teaching objectives, training content, communication skills, etc, (2) Improve ACP-related knowledge and ability of medical staff or medical students, (3) Increase public awareness and participation in the importance of ACP, (4) Promote doctor-patient consensus on end-of-life decisions.

#### Context.

ACP workshops in medical institutions, (2) ACP public training organized by the Community Health Service Center, (3) ACP education programs for nursing homes or hospice teams, (4) ACP teaching in university medical/nursing schools.

Studies will excluded if (1) the population included in the study had an age of < 18 years; (2) introduce the concept of ACP, does not involve specific workshop content; (3) Workshops are used for consensus content.

We will use the reference management software EndNote X9 to download abstracts from each database to deduplicate search results [32]. To ensure the consistency and reliability of the study selection process, all team members will meet before the screening process to ensure the team is aligning in their understanding of the inclusion criteria. First, two reviewers will independently screen titles and abstracts against the inclusion and exclusion criteria. Next, the same two reviewers who screened the titles and abstracts will read the full text to screen full-text articles. Consensus by the third reviewer will resolve any disagreement regarding inclusion or exclusion. Any full-text articles excluded after screening will include the reasoning behind exclusion. A PRISMA flowchart will summarise the search, screening, and identification process for relevant studies [29], depicted in S5 Fig.

In the process of screening and including studies, we will pay attention to assessing whether the selected studies have cultural biases or potential ethical issues. This includes, but is not limited to, ensuring the privacy and confidentiality of secondary data, ensuring that the interpretation and analysis of the data are fair, objective, and comprehensive, and ensuring that data sources are clearly and accurately cited and acknowledged in the results.

During this phase, the quality of the included studies will be carefully assessed using the Mixed Methods Appraisal Tool (MMAT) [33]. Specifically, When extracting data from the included studies, we will document the MMAT quality scores for each study. In our data analysis, we will give more weight to studies with higher quality scores, meaning that findings from methodologically robust studies will have a greater influence on our conclusions. Furthermore, when interpreting the results, we will thoroughly discuss the implications of the quality differences among the studies. We will highlight the strengths and weaknesses of each study and assess how these factors may affect the generalizability and reliability of our findings. All studies will be included in the final analysis to provide a comprehensive overview of the existing evidence, reflecting the inclusive nature of a scoping review. However, we will clearly acknowledge and discuss the methodological quality of each study in our results and conclusions.

### Step 4: Data extraction

Data will extracted from the selected articles by two independent reviewers (LY and CS) using a data-charting table in duplicate Microsoft Excel that will approved by the research team. We will resolve any disagreement through discussion until a consensus is reached.

The extracted data included the following items:(1) Study characteristics (i.e., authors, year of publication, and study design); (2) Information related to the sampled population (i.e., type, number); (3) The workshop (i.e., its main components, duration, format, and the providers involved); (4) The targeted outcomes (including trainees’ Knowledge, attitude, comfort, confidence, behavior, and readiness of ACP); (5) The measurement tools and the main results.

The extracted information will be presented in tabular form in S6 Table, and the tables will be amended as the scoping review progresses. To enhance the quality and accuracy of the data, a third reviewer will review and verify all extracted information. This comprehensive data collection process will be anticipated to extend over approximately three months.

### Step 5: Data synthesis and presentation of results

We will report on the study selection process, the characteristics of the included studies, the population characteristics, the workshop’s content, and the primary results. Insights from the quality assessment will be incorporated throughout this analysis to provide a nuanced understanding of the findings. Each research question will be answered using a quantitative summary that provides an overview of the quantity and types of included literature.

To address the heterogeneity among studies, particularly in terms of their designs, populations, and interventions, we will employ a structured approach. Specifically, we will categorize studies based on their methodological designs, such as randomized controlled trials, cohort studies, and qualitative studies, and analyze them separately. Where feasible, for quantitative studies with sufficient homogeneity, we will employ meta-analysis. For qualitative research, thematic synthesis will be conducted. We will perform a group analysis based on the characteristics of the study population, including demographics, clinical conditions, and environment, to explore the effectiveness of ACP workshops across different populations. We will detail and compare the content and delivery methods of ACP workshops, assess the consistency of intervention components across studies, and explore the impact of different intervention elements on outcomes. Where feasible, we will attempt to isolate the effects of specific intervention components through sensitivity or subgroup analyses.

To enhance clarity and facilitate understanding, the effects of ACP workshops in different dimensions will be visually displayed through charts, tables, and other forms. Specifically, we will employ a comprehensive narrative synthesis approach, complemented by detailed data analysis methods, such as meta-analysis,thematic synthesis, and sensitivity or subgroup analyses, highlighting the impact of study quality on our interpretations.

All investigator members of the team will be involved in discussing not only the final results and implications but also the implications of the quality assessment on our understanding of the evidence base. The final report, following the PRISMA-ScR guidelines [29], will transparently present the findings, incorporating the critical appraisal of the included studies as a key component of our comprehensive review.

### Step6: Stakeholder consultation

To ensure the relevance and applicability of our findings, we will engage in a stakeholder consultation process, involving researchers with extensive experience in ACP workshop training. Specifically, we will present our scoping review findings to these stakeholders in a structured format, firstly, through a detailed report or presentation, outlining each finding item by item. Subsequently, we will invite the experts to review these results and provide their valuable feedback. Following the stakeholder consultation, we will revise and refine the report based on the issues and suggestions raised in the feedback. The modifications may include improving content organization and supplementing additional data.

## Discussion

ACP workshop, as a pivotal strategy to foster doctor-patient communication [20], has increasingly gained prominence. However, despite the potential demonstrated by ACP workshops in improving participants’ understanding, skills, and confidence in ACP [17–20], the current literature lacks comprehensive information on the diversity and effectiveness of these workshops. Specifically, there is a dearth of systematic comparisons and syntheses regarding how different types of ACP workshops impact various target groups, such as healthcare professionals, medical students, and the general public, as well as their long-term effects on patient care quality and healthcare resource utilization. Consequently, this study aims to systematically map the existing literature through a scoping review methodology, providing a comprehensive overview of ACP workshops and identifying knowledge gaps to guide future research directions and practical applications.

This study employs the scoping review method, adhering to the JBI guidelines and the PRISMA-ScR framework [26–28], to ensure systematicity and comprehensiveness. This approach is well-suited for exploring the literature in the field of ACP workshops, as it enables the systematic collection, screening, extraction, and synthesis of existing research, thereby offering a detailed overview of ACP workshops [25]. Specifically, we employed a comprehensive search strategy utilizing the broad search terms ‘advance care planning’ and ‘workshop,’ along with their synonyms and related phrases, across multiple databases and platforms, and over a specified time range, to capture all relevant studies and minimize the risk of publication bias. Additionally, stringent and explicit inclusion and exclusion criteria were established, characterized by objectivity and reproducibility [27], to ensure that only studies meeting our predefined standards are included in the review. To further reduce the risk of selection bias, two reviewers will independently screen studies and resolve any discrepancies in study selection through discussion and consensus, ensuring an unbiased evaluation of each study. Furthermore, the study selection process, including reasons for inclusion and exclusion, will be documented in detail to ensure the transparency and reproducibility of our findings. Although scoping reviews typically do not involve detailed assessments of study quality, we have decided to use the Mixed Methods Appraisal Tool (MMAT) to evaluate the quality of the included literature [33]. A data extraction template has also been developed to standardize the data extraction process and minimize potential data extraction bias.

However, several limitations are acknowledged that may impact the generality of our findings. Firstly, the diversity and complexity of ACP workshops introduce a significant challenge to our data analysis. This variability in the quality and content of the related literature poses potential biases and heterogeneity, which could affect the validity and reliability of our conclusions. The inconsistent nature of ACP workshop implementations may limit our ability to draw broad, universal conclusions from the data. Secondly, the decision to include only English articles introduces a language bias, that may overshadow significant studies published in other languages. This language barrier not only restricts our dataset but also potentially underestimates the cultural nuances and differences in ACP practices across diverse cultural contexts. By focusing solely on English literature, we may miss crucial insights from non-English-speaking regions, thereby limiting the cross-cultural applicability of our findings. Lastly, the preponderance of studies focusing on short-term effects of ACP workshops presents another limitation. This emphasis on immediate outcomes restricts our understanding of the long-term impact and sustainability of ACP interventions. The lack of longitudinal studies may hinder our ability to assess the enduring effects of ACP workshops on patient care and end-of-life decision-making. During data extraction and synthesis, we will carefully consider these factors and strive to account for potential biases, acknowledge the limitations imposed by language and cultural constraints, and highlight the need for future research to address these gaps, particularly in the areas of cross-cultural ACP practices and long-term outcomes.

The findings of this review hold significant implications for ACP across medical education, clinical practice, and policy development. They offer the potential to enrich participants’ learning experiences, boost the implementation of ACP, and ultimately enhance patient decision-making, improve hospice care quality, and facilitate the efficient allocation and utilization of medical resources. Despite inherent limitations, this review lays a solid foundation for future research and practice in the realm of ACP workshops.

Future research could delve deeper into the effectiveness of specific types of ACP (Advance Care Planning) workshops by examining which formats, durations, and content are most beneficial for different participant groups. Additionally, cross-cultural comparative studies should be conducted to explore how ACP workshops are perceived and utilized in diverse cultural contexts, identifying any cultural-specific factors that may influence their effectiveness. Furthermore, long-term follow-up research is necessary to assess the sustained impact of ACP workshops on participants’ understanding, attitudes, and behaviors related to advance care planning, as well as to explore any potential changes in healthcare outcomes. These comprehensive approaches will enrich our understanding and application of ACP workshops.

## Supporting information

S1 Checklist The PRISMA-ScR checklist. (DOCX)

S2 Checklist The PRISMA-P 2015 checklist. (DOCX)

S3 Table The search strategy for PubMed. (DOCX)

S4 File The Mixed Methods Appraisal Tool (MMAT). (PDF)

S5 Fig PRISMA flowchart. (DOCX)

S6 Table The data extraction table template. (DOCX)

## References

[1] Sudore RL, Lum HD, You JJ, Hanson LC, Meier DE, Pantilat SZ, et al. Defining advance care planning for adults: a consensus definition from a multidisciplinary delphi panel. J Pain Symptom Manage. 2017;53(5): 821-832.e1. doi: 10.1016/j.jpainsymman.2016.12.331 28062339 PMC5728651

[2] Rietjens JAC, Sudore RL, Connolly M, van Delden JJ, Drickamer MA, Droger M, et al. Definition and recommendations for advance care planning: an international consensus supported by the European Association for Palliative Care. Lancet Oncol. 2017;18(9):e543–e551. doi: 10.1016/S1470-2045(17)30582-X 28884703

[3] Chikada A, Takenouchi S, Nin K, Mori M. Definition and recommended cultural considerations for advance care planning in Japan: a systematic review. Asia Pac J Oncol Nurs. 2021;8(6):628–638. doi: 10.4103/apjon.apjon-2137 34790847 PMC8522591

[4] Fleuren N, Depla MFIA, Janssen DJA, Huisman M, Hertogh CMPM. Underlying goals of advance care planning (ACP): a qualitative analysis of the literature. BMC Palliat Care. 2020;19(1):27. doi: 10.1186/s12904-020-0535-1 32143601 PMC7059342

[5] Liu X, Ho M-H, Wang T, Cheung DST, Lin C-C. Effectiveness of dyadic advance care planning: a systematic review and meta-analysis. J Pain Symptom Manage. 2024;67(6):e869–e889. doi: 10.1016/j.jpainsymman.2024.01.027 38272378

[6] Gomes Souza L, Bouba DA, Corôa R de C, Dofara SG, Robitaille V, Blanchette V, et al. The impact of advance care planning on healthcare professionals’ well-being: a systematic review. J Pain Symptom Manage. 2024;67(2):173–187. doi: 10.1016/j.jpainsymman.2023.09.026 37827454

[7] Nguyen K-H, Sellars M, Agar M, Kurrle S, Kelly A, Comans T. An economic model of advance care planning in Australia: a cost-effective way to respect patient choice. BMC Health Serv Res. 2017;17(1):797. doi: 10.1186/s12913-017-2748-4 29191183 PMC5709848

[8] Goswami P. Impact of advance care planning and end-of-life conversations on patients with cancer: an integrative review of literature. J Nurs Scholarsh. 2023;55(1): 272–290. doi: 10.1111/jnu.12804 35946931

[9] Lenko RA, Hoffman GJ, Robinson-Lane SG, Silveira MJ, Voepel-Lewis T. Achieving goal-concordant care: formal and informal advance care planning for White, Black, and Hispanic older adults. J Am Geriatr Soc. 2024;72(8):2412–2422. doi: 10.1111/jgs.18971 38760957 PMC11323214

[10] Wilkin K, Fang ML, Sixsmith J. Implementing advance care planning in palliative and end of life care: a scoping review of community nursing perspectives. BMC Geriatr. 2024;24(1):294. doi: 10.1186/s12877-024-04888-4 38549045 PMC10976700

[11] Inokuchi R, Hanari K, Shimada K, Iwagami M, Sakamoto A, Sun Y, et al. Barriers to and facilitators of advance care planning implementation for medical staff after the COVID-19 pandemic: an overview of reviews. BMJ Open. 2023;13(10):e075969. doi: 10.1136/bmjopen-2023-075969 37816562 PMC10565150

[12] Canny A, Mason B, Boyd K. Public perceptions of advance care planning (ACP) from an international perspective: a scoping review. BMC Palliat Care. 2023;22(1):107. doi: 10.1186/s12904-023-01230-4 37507777 PMC10375610

[13] Stevens J, Elston D, Tan A, Barwich D, Carter RZ, Cochrane D, et al. Clinicians’ experiences implementing an advance care planning pathway in two Canadian provinces: a qualitative study. BMC Prim Care. 2024;25(1): 217. doi: 10.1186/s12875-024-02468-4 38879532 PMC11179357

[14] Zhu N, Yang L, Wang X, Tuo J, Chen L, Deng R, et al. Experiences and perspectives of healthcare professionals implementing advance care planning for people suffering from life-limiting illness: a systematic review and meta-synthesis of qualitative studies. BMC Palliat Care. 2023;22(1):55. doi: 10.1186/s12904-023-01176-7 37149560 PMC10163819

[15] Mukurunge E, Reid M, Fichardt A, Nel M. Interactive workshops as a learning and teaching method for primary healthcare nurses. Health SA. 2021;26:1643. doi: 10.4102/hsag.v26i0.1643 34956654 PMC8678960

[16] Forsetlund L, O’Brien MA, Forsén L, Reinar LM, Okwen MP, Horsley T, et al. Continuing education meetings and workshops: effects on professional practice and healthcare outcomes. Cochrane Database Syst Rev. 2021;9(9):CD003030. doi: 10.1002/14651858.CD003030.pub3 34523128 PMC8441047

[17] Vellani S, Maradiaga Rivas V, Nicula M, Lucchese S, Kruizinga J, Sussman T, et al. Palliative approach to care education for multidisciplinary staff of long-term care homes: a pretest post-test study. Gerontol Geriatr Med. 2023;9:23337214231158470. doi: 10.1177/23337214231158470 36845318 PMC9947670

[18] Pan CX, Spinelli A, Litrivis E, Popoviciu A, Thomson KP, Brondolo E. AD-LAST! An interdisciplinary clinical workshop to improve cultural and spiritual awareness in advance care planning skills. Palliat Support Care. 2023;21(3):422–428. doi: 10.1017/S1478951522000232 35289264

[19] Carter RZ, Siden E, Husband A, Barwich D, Soheilipour S, Kryworuchko J, et al. Community-led, peer-facilitated Advance Care Planning workshops prompt increased Advance Care Planning behaviors among public attendees. PEC Innov. 2023;3:100199. doi: 10.1016/j.pecinn.2023.100199 37662691 PMC10474229

[20] Grey C, Constantine L, Baugh GM, Lindenberger E. Advance Care Planning and shared decision-making: an interprofessional role-playing workshop for medical and nursing students. MedEdPORTAL. 2017;13:10644. doi: 10.15766/mep_2374-8265.10644 30800845 PMC6338169

[21] Blomberg BA, Quintana C, Hua J, Hargis-Fuller L, Laux J, Drickamer MA. Enhancing Advance Care Planning communication: an interactive workshop with role-play for students and primary care clinicians. MedEdPORTAL. 2020;16:10973. doi: 10.15766/mep_2374-8265.10973 32964122 PMC7499812

[22] Rabow MW, McGowan M, Small R, Keyssar R, Rugo HS. Advance Care Planning in community: an evaluation of a Pilot 2-session, nurse-led workshop. Am J Hosp Palliat Care. 2019;36(2):143–146. doi: 10.1177/1049909118797612 30153741

[23] McDermott E, Selman LE. Cultural factors influencing advance care planning in progressive, incurable disease: a systematic review with narrative synthesis. J Pain Symptom Manage. 2018;56(4):613-636. doi: 10.1016/j.jpainsymman.2018.07.006 30025936

[24] McMahan RD, Tellez I, Sudore RL. Deconstructing the complexities of advance care planning outcomes: what do we know and where do we go? A scoping review. J Am Geriatr Soc. 2021;69(1):234–244. doi: 10.1111/jgs.16801 32894787 PMC7856112

[25] Tricco AC, Lillie E, Zarin W, O’Brien K, Colquhoun H, Kastner M, et al. A scoping review on the conduct and reporting of scoping reviews. BMC Med Res Methodol. 2016;16:15. doi: 10.1186/s12874-016-0116-4 26857112 PMC4746911

[26] Lockwood C, Dos Santos KB, Pap R. Practical guidance for knowledge synthesis: scoping review methods. Asian Nurs Res (Korean Soc Nurs Sci). 2019;13(5): 287–294. doi: 10.1016/j.anr.2019.11.002 31756513

[27] Peters MDJ, Marnie C, Tricco AC, Pollock D, Munn Z, Alexander L, et al. Updated methodological guidance for the conduct of scoping reviews. JBI Evid Implement. 2021;19(1):3–10. doi: 10.1097/XEB.0000000000000277 33570328

[28] Peters MDJ, Godfrey C, McInerney P, Khalil H, Larsen P, Marnie C, et al. Best practice guidance and reporting items for the development of scoping review protocols. JBI Evid Synth. 2022;20(4):953–968. doi: 10.11124/JBIES-21-00242 35102103

[29] Tricco AC, Lillie E, Zarin W, O’Brien KK, Colquhoun H, Levac D, et al. PRISMA Extension for Scoping Reviews (PRISMA-ScR): checklist and explanation. Ann Intern Med. 2018;169(7):467–473. doi: 10.7326/M18-0850 30178033

[30] Shamseer L, Moher D, Clarke M, Ghersi D, Liberati A, Petticrew M, et al. Preferred reporting items for systematic review and meta-analysis protocols (PRISMA-P) 2015: elaboration and explanation. BMJ. 2015;350:g7647. doi: 10.1136/bmj.g7647 25555855

[31] McGowan J, Sampson M, Salzwedel DM, Cogo E, Foerster V, Lefebvre C. PRESS Peer Review of Electronic Search Strategies: 2015 guideline statement. J Clin Epidemiol. 2016;75:40–46. doi: 10.1016/j.jclinepi.2016.01.021 27005575

[32] Bramer WM, Giustini D, de Jonge GB, Holland L, Bekhuis T. De-duplication of database search results for systematic reviews in EndNote. J Med Libr Assoc. 2016;104(3):240–243. doi: 10.3163/1536-5050.104.3.014 27366130 PMC4915647

[33] Hong QN, Gonzalez-Reyes A, Pluye P. Improving the usefulness of a tool for appraising the quality of qualitative, quantitative and mixed methods studies, the Mixed Methods Appraisal Tool (MMAT). J Eval Clin Pract. 2018;24(3):459–467. doi: 10.1111/jep.12884 29464873

