# Supplementary material for: Effects of different types of Advance Care Planning workshops: A scoping review protocol
Source: PLoS One. 2025 May 20;20(5):e0322661. doi: 10.1371/journal.pone.0322661 (PMC12091826; doi:10.1371/journal.pone.0322661)
Supplement: S3 Table — (DOCX) [file pone.0322661.s003.docx]

**Supporting information 1**

**Search strategy for PubMed on 8/8/2024**

| Code | Search Strategy | Results |
| --- | --- | --- |
| #1 | "Advance Care Planning"[Mesh] | 11,639 |
| #2 | advance care planning[Title/Abstract] | 5,476 |
| #3 | #1 OR #2 | 13,638 |
| #4 | "Education"[Mesh] | 923,956 |
| #5 | (Workgroup[Title/Abstract]) OR (workshop[Title/Abstract]) | 39,713 |
| #6 | #4 OR #5 | 955,665 |
| #7 | #3 AND #6 | 1,153 |
