# Supplementary material for: Effects of different types of Advance Care Planning workshops: A scoping review protocol
Source: PLoS One. 2025 May 20;20(5):e0322661. doi: 10.1371/journal.pone.0322661 (PMC12091826; doi:10.1371/journal.pone.0322661)
Supplement: S6 Table — (DOCX) [file pone.0322661.s006.docx]

**Supporting information 2**

**Data extraction table template**

| Study | Year | Country | Design | Workshop | | | | | | | Outcomes |
| --- | --- | --- | --- | --- | --- | --- | --- | --- | --- | --- | --- |
|  |  |  |  | Aims | Settings | Participants | Procedure | Lasted time | Topics | Educational materials |  |
|  |  |  |  |  |  |  |  |  |  |  |  |
|  |  |  |  |  |  |  |  |  |  |  |  |
|  |  |  |  |  |  |  |  |  |  |  |  |
|  |  |  |  |  |  |  |  |  |  |  |  |
